# Regulatory T cells and macrophages in atherosclerosis: from mechanisms to clinical significance

**DOI:** 10.3389/fimmu.2024.1435021

**Published:** 2024-11-08

**Authors:** Xin Ouyang, Zhongyong Liu

**Affiliations:** ^1^ Clinical Medical College, Jiangxi University of Chinese Medicine, Nanchang, China; ^2^ Department of Cardiology, Affiliated Hospital of Jiangxi University of Chinese Medicine, Nanchang, China

**Keywords:** cardiovascular disease, atherosclerosis, regulatory T cells, Tregs, macrophages

## Abstract

Atherosclerosis is a complex pathological process, which causes diseases that threaten the health of an increasing number of people. Studies have found that the original view of lipid accumulation is not comprehensive because the use of lipid-lowering drugs alone cannot effectively treat atherosclerosis. As the study of the pathogenesis of atherosclerosis develops in-depth, the impact of immune-inflammatory response on atherosclerosis has garnered a great deal of attention. Some new advances have been made in the role of regulatory T cells (Tregs) and macrophages with unique immunomodulatory functions in atherosclerosis. Herein, the role of Tregs, macrophages, the mechanisms of Tregs-regulated macrophages, and the effects of potential factors on Tregs and macrophages in atherosclerosis are overviewed. Targeting Tregs and macrophages may provide new research strategies for the treatment of atherosclerosis in the clinic.

## 1 Introduction

Atherosclerosis is the pathologic basis of multiple cardio-cerebral vascular diseases such as ischemic cardiomyopathy, myocardial infarction, ischemic stroke, and peripheral arterial disease (1, 2). Nowadays, atherosclerosis involves a wide range of groups, and the onset tends to be younger (2), which has attracted extensive attention from academics and has been the subject of long-term research.

In the past, atherosclerosis was considered a disease of cholesterol accumulation, which was caused by the retention of lipoproteins in the intimal of arteries (3). Unfortunately, cholesterol-lowering treatments with drugs like statins don’t even prevent 70% of clinical atherosclerotic events (4). In recent years, it has been recognized that atherosclerosis is a chronic immune-inflammatory disease characterized by the accumulation of immune cells and lipids in the vascular wall (5). A randomized controlled trial showed that without affecting lipid levels, anti-inflammatory treatment led to a significant reduction in the recurrence of cardiovascular events (4), demonstrating the important impact of inflammation on atherosclerosis. The macrophages and regulatory T cells (Tregs) among immune cells are valued as classical promoters and suppressors of atherosclerosis, and they are jointly involved in the immune-inflammatory response in atherosclerosis. Several studies have shown that innate and adaptive immune responses are activated in the process of atherosclerosis (6–8). The innate immunity depends largely on macrophages and abundant pro-inflammatory cytokines secreted upon activated macrophages, including interleukin (IL)-1β, tumor necrosis factor (TNF)-α, and chemokines such as C-C motif chemokine ligand (CCL) 2, CCL5 (2, 9). They are the central cells in the atherosclerotic process (10). In the adaptive immunity, T cell-mediated immune responses, especially the modulation of atherosclerosis through Tregs-mediated inflammatory signaling, have attracted considerable enthusiasm from researchers. Numerous studies have shown that Tregs are atheroprotective (2, 11, 12).

This review will systematically and comprehensively summarize the current findings on the role of Tregs and macrophages in atherosclerosis, focusing more on how Tregs regulate macrophages and the effects of underlying factors on Tregs and macrophages.

## 2 Role of macrophages in atherosclerosis

**Figure 1:**
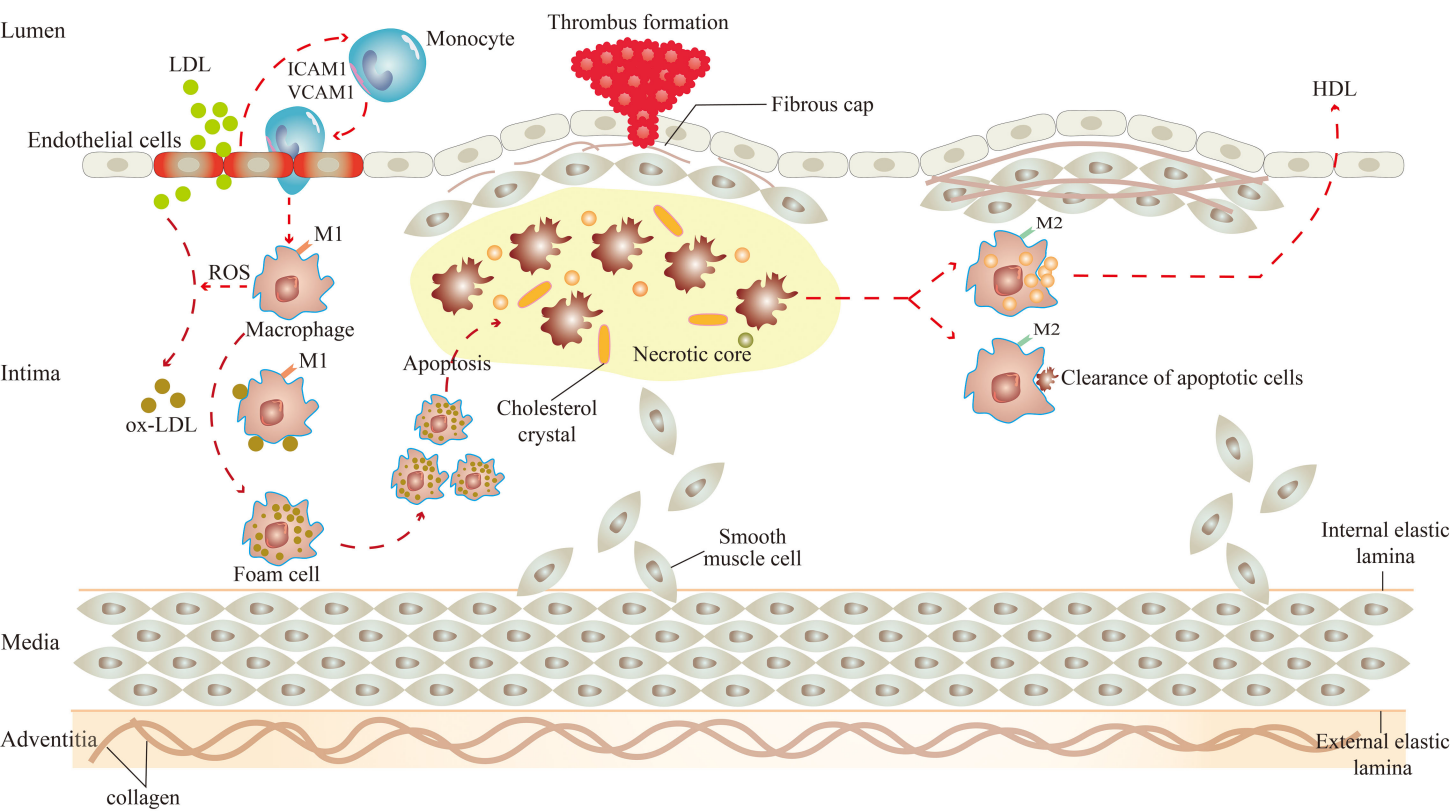
Role of macrophages in atherosclerosis. There are two subsets of macrophages, M1 and M2 macrophages, and they function at different stages of atherosclerosis. In the initiation of atherosclerosis, M1 macrophages engulf ox-LDL to become foam cells. In the progression of atherosclerosis, M1 macrophages-derived foam cells undergo apoptosis, which leads to the formation of necrotic cores, and eventually to the progression of plaque rupture and thrombosis. In the regression of atherosclerosis, M2 macrophages promote high cholesterol efflux and clear apoptotic cells by efferocytosis.

Macrophages play a crucial role in the development of atherosclerosis and are involved throughout the process of atherosclerosis, including the initiation and progression of atherosclerotic lesions, the advanced necrotic lesions, and the regression and resolution of lesions (13) (**Figure 1**).

Under the influence of various atherosclerotic risk factors (smoking, hypertension, hyperlipidemia, etc.), vascular endothelial cells are damaged (14). At this time, excessive low-density lipoprotein (LDL) in the blood can cross the gap between endothelial cells to enter the intima (15). LDL, in turn, activates endothelial cells, expressing cell adhesion molecules such as intercellular adhesion molecule 1 (ICAM1) and vascular cell adhesion molecule 1 (VCAM1) to capture monocytes adhesion to endothelial cells (15). Subsequently, monocytes enter the arterial vessel wall and differentiate into macrophages (15).

The effects of macrophages on atherosclerosis are inextricably linked to their polarization and the resulting phenotype (16). Activated macrophages are broadly classified into two subsets, M1 and M2 macrophages (17). Significantly, the two subsets of macrophages are activated by different factors. M1 macrophages arise in the presence of cytokines like interferon (IFN)-γ and IL-12, and M2 macrophages arise from stimuli such as IL-10 and transforming growth factor (TGF)-β. Consequently, they have their own characteristics and functions in atherosclerosis.

There are two main roles for M1 macrophages in atherosclerosis. On the one hand, they can induce tissue damage. M1 macrophages can activate the nicotinamide adenine dinucleotide phosphate (NADPH) oxidase complex, leading to the production of reactive oxygen species (ROS) (18). Under the action of ROS, the LDL is oxidatively modified to become oxidized LDL (ox-LDL). Then, the surface receptors on macrophages quickly identify ox-LDL and engulf them to transform into foam cells, which is the initiation of atherosclerotic lesions (19). Meanwhile, M1 macrophages express chemokine receptor ligands to promote the recruitment of helper T cell (Th)1 and natural killer (NK) cells, resulting in a sustained inflammatory response (20), which damages surrounding tissues in the aseptic environment of atherosclerosis (21). On the other hand, they can lead to plaque rupture. Macrophage-derived foam cells secrete cytokines, which make the site more vulnerable to inflammation and instigate smooth muscle cell proliferation (22, 23). If the local pro-inflammatory microenvironment continues, monocyte infiltration, macrophage apoptosis, and defective clearance of apoptotic cells increase (24, 25), producing atherosclerotic plaques with large necrotic cores and thin fibrous caps (15). The initial calcium deposition with M1 macrophage within the necrotic core of the lesions is called microcalcification, which is related to plaque rupture (26). Once the plaque ruptures, pro-thrombotic substances in the plaque are exposed, inducing platelet activation, thrombus formation, and arterial occlusion (27). M2 macrophages also play two major roles in atherosclerosis. In the first place, they stabilize plaque. The proportion of M2 macrophages in stable plaques is relatively higher than in M1 macrophages (28). In contrast to the M1 macrophage, the calcium deposition with M2 macrophages is called macrocalcification, leading to plaque stability (26). And in the second place, they regress plaque. A study by Rahman et al. showed that the increased markers of M2 macrophages were consistent with the regression of atherosclerotic plaque. Therefore, it can be considered that the polarization of macrophage to the M2 state was necessary for the regression of atherosclerotic inflammation and plaque (29). These effects are closely linked to the efflux and pro-resolving of macrophages. It was reported that M2 macrophages can promote the efflux of high cholesterol from lipid-laden macrophages, and clear apoptotic cells by enhancing the efferocytosis in atherosclerotic lesions (15, 30).

Due to the heterogeneity and plasticity of macrophages (31), M2 macrophages can be further classified into four different subsets: M2a, M2b, M2c, and M2d macrophages. Interestingly, the differentiation of M2 subtypes has been associated with different stimuli (32). M2a macrophages are induced by IL-4 and IL-13. M2b macrophages are polarized by immune-complex, such as Toll-like receptor (TLR) ligands and IL-1 receptor agonists. M2c macrophages are activated by IL-10 and glucocorticoids. M2d macrophages are stimulated by IL-6 or co-stimulation with TLR and adenosine A_2A_ receptor agonists (16, 32–34). These macrophages play a role in atherosclerosis. M2a macrophages have been described as “wound-healing” or “tissue-repairing” macrophages, which promote the deposition of extracellular matrix by the release of TGF-β (32, 35). M2b and M2c macrophages are known as “regulatory macrophages”, which share immunoregulatory functions in atherosclerosis (16). Both of them can release IL-10, suppress pro-inflammatory cytokines such as IL-12, function as antigen presenting cells, and retain the ability to produce pro-inflammatory cytokines such as IL-1β, IL-6, and TNF (32). In addition, M2c macrophages exhibit high levels of the Mer tyrosine kinase (MerTK) that gives them high efferocytosis capacity (34, 36). M2d macrophages express high levels of IL-10 and vascular endothelial growth factor (VEGF), and low levels of TNF and IL-12, contributing to angiogenesis and resistance to lipid accumulation (16, 34). Notably, an increasing number of macrophage phenotypes are being recognized, such as hemoglobin-related phenotypes (Mhem), redox-regulatory metabolic (Mox), and M4 macrophages (37). Mhem macrophages have atheroprotective effects, which phagocytize excess cholesterol to make this phenotype resistant to acquiring a foam cell phenotype (38). Contrary to Mhem macrophages, Mox macrophages have proatherogenic effects, resulting in foam cell formation by ox-LDL accumulation (38). M4 macrophages have defective phagocytic capacities and high productions of pro-inflammatory cytokines, which detrimentally contribute to plaque development (39, 40).

## 3 Function of regulatory T cells in atherosclerosis

Tregs, a subpopulation of T lymphocytes with unique immunoregulatory functions, are closely related to the pathogenesis of atherosclerosis. According to their developmental origin, Tregs can be mainly divided into two classifications. One is naturally generated in the thymus, called tTregs, and the other is differentiated from the periphery, called pTregs (41, 42). pTregs are mainly induced from naïve CD4^+^ T cells after they are stimulated by antigens in peripheral lymphoid organs, while Foxp3^+^CD4^+^ tTregs are developed from CD4^+^CD25^+^ T cells in the thymus (43). The majority of the studies focus on the canonical CD4^+^CD25^+^Foxp3^+^ Treg phenotype (44). It refers to the CD4^+^ T cells with high expression of CD25. In their nucleus, the transcription factor FOXP3 is expressed specifically, which acts as a critical regulator of Treg immunosuppression by upregulating the expression of other surface molecules such as CD25 and CTLA-4 (45).

**Figure 2:**
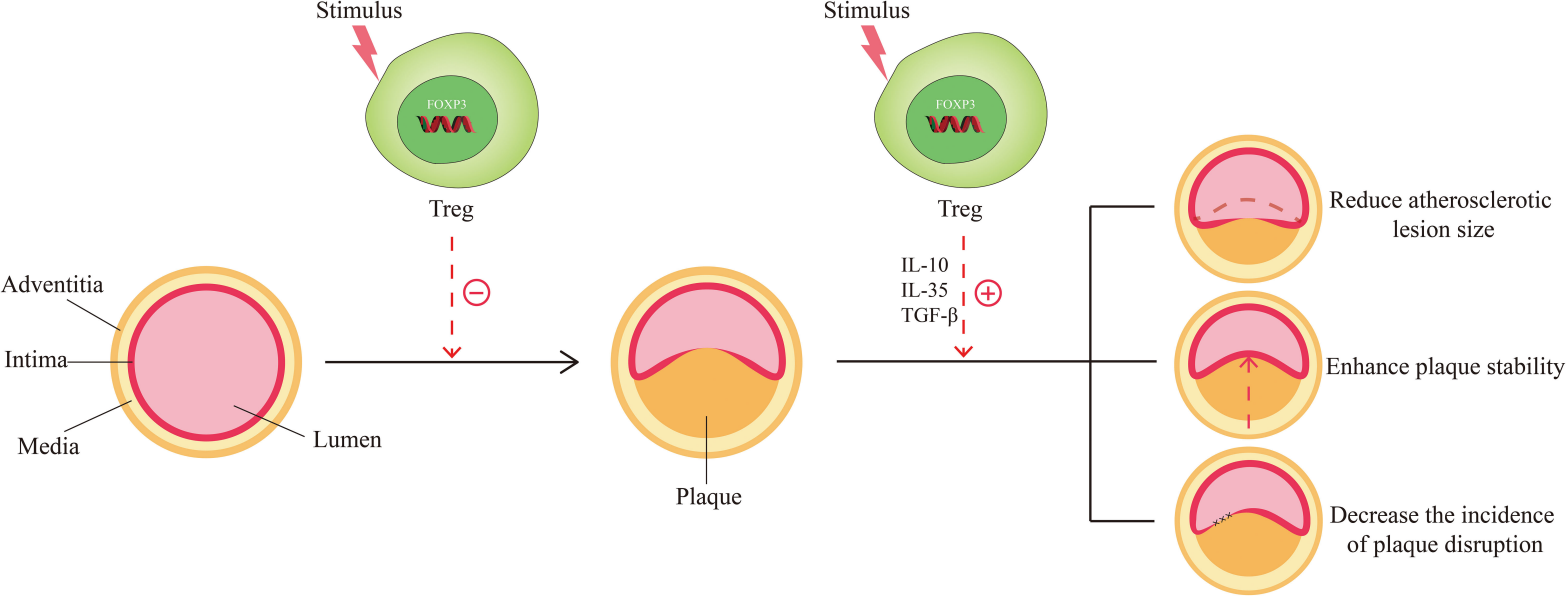
Function of Tregs in Atherosclerosis. The normal arteries are composed of three layers: the adventitia, the media, and the intima. When stimulated by risk factors (smoking, hypertension, hyperlipidemia, etc.), normal arteries undergo abnormal changes and move toward atherosclerosis. Under these circumstances, Tregs are stimulated and activated, which in turn exert the effect of countering the formation of atherosclerosis. When atherosclerosis develops, activated Tregs could function in response to the size, stability, and disruption of the plaque by secreting inhibitory cytokines such as IL-10, IL-35, and TGF-β, thereby favoring disease improvement.

The role of Tregs in atherosclerosis has been reported successively. Increasing evidence has shown that Tregs mediate immunomodulation and protect against atherosclerosis. Specifically, there are three implications. From the process of atherogenesis, Tregs can hinder its progression (**Figure 2**). Studies have found that changes in the number of Tregs determine changes in atherosclerotic activity. Ait-Oufella et al. unraveled that a deficiency of Tregs was associated with the development of atherosclerosis and promoted the progression of atherosclerotic lesions (46). Ou XM et al. proved that the Huxin formula, a formula of Chinese medicine, may exert its anti-atherosclerotic effects by increasing counts of Tregs to restrain inflammatory response, reduce inflammatory cell infiltration, and attenuate aortic root fibrosis (47). Wang F et al. indicated that dendritic cell-expressed IDO can induce aortic CD4^+^CD25^+^Foxp3^+^ Treg expansion through the IDO-Kyn-AHR axis to reduce atherosclerotic lesions (48). de Boer et al. found that low numbers of FOXP3^+^ Tregs were present in all developmental stages of human atherosclerotic lesions (49). George et al. also found Tregs were reduced in patients with vulnerable coronary plaques (50). In terms of plaque characteristics, Tregs can reduce the size of the atherosclerotic lesion, enhance the stability of plaque, and decrease the incidence of plaque rupture (**Figure 2**). Mor A. et al. found that the number of Tregs was reduced in atherosclerotic apolipoprotein E-deficient/knockout (ApoE^-/-^) mice (11). Yet, the transfer of wild-type Tregs into ApoE^-/-^ mice resulted in a significant reduction in aortic sinus plaque compared to controls (11). Further evidence was provided by the study of Klingenberg et al., and they concluded from animal experiments that the depletion of Tregs led to a 2.1-fold increase in the size of atherosclerotic plaque (51). On the contrary, the adoptive transfer of Tregs to ApoE^-/-^ mice altered the composition of plaques so that plaques exhibited a tendency toward a more stable phenotype, thereby lowering the incidence of plaque rupture (52). An T et al. detected that soluble fibrinogen-like protein 2 (sFgl2), a novel effector of Tregs, reduced plaque area and enhanced plaque stability mainly by forming a positive feedback pathway with Tregs, increasing the abundance and immunosuppressive function of Tregs (53). From the perspective of Tregs themselves, the inhibitory cytokines such as IL-10, IL-35, and TGF-β can be secreted by them to suppress the immune-inflammatory response of atherosclerosis. IL-10 has anti-atherosclerotic effects and affects not only plaque formation but also plaque size and stability (54, 55). TGF-β can inhibit the recruitment and activation of inflammatory cells in atherosclerotic plaque and promote the proliferation and survival of smooth muscle cells and collagen biosynthesis to increase the stability of plaques and ameliorate atherosclerosis (56, 57). IL-35 inhibits the proliferation of T cells, regulates the activation of naïve T cells, and inhibits the production of pro-inflammatory factors, thus preventing the development of atherosclerosis (58, 59).

## 4 Regulation of macrophages by regulatory T cells in atherosclerosis

### 4.1 Tregs deficiency induces the accumulation of macrophages to aggravate atherosclerotic lesions

**Figure 3:**
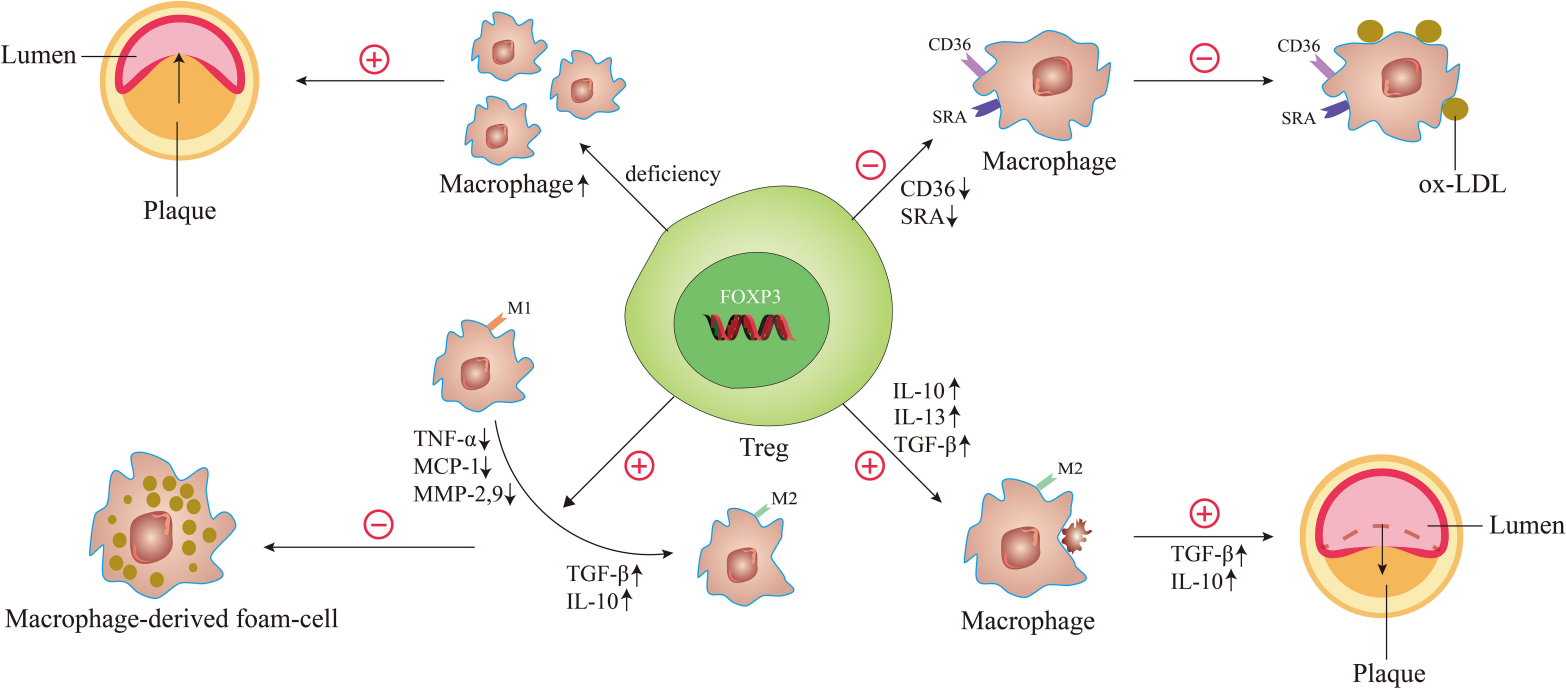
The underlying mechanisms of Tregs regulate macrophages in atherosclerosis. When the number of Tregs is decreased and the function is reduced, it can cause the accumulation of macrophages, leading to the acceleration of atherosclerosis and the decrease of plaque stability. When Tregs are activated, the expression of scavenger receptor SRA and CD36 is decreased, thereby reducing oxidized LDL uptake of macrophages. Activated Tregs facilitate the differentiation of M1 macrophages to M2 macrophages by decreasing the secretion of pro-inflammatory factors such as TNF-α, MCP-1, and MMP-9 and increasing the secretion of anti-inflammatory factors such as TGF-β and IL-10, thereby inhibiting the formation of macrophage-derived foam-cell. During atherosclerosis regression, the Treg-derived cytokines such as IL-13, IL-10, and TGF-β enhance the efferocytosis capacity of macrophages by promoting M2 macrophages secretion of IL-10 and TGF-β.

In 2006, Ait-Oufella et al. demonstrated for the first time that endogenous CD4^+^CD25^+^Tregs have a protective role in atherogenesis. To illustrate it, they used CD25-specific antibody that depleted Tregs to treat ApoE^-/-^ mice. It turned out that the size of the atherosclerotic lesion was increased by 50% and the accumulation of macrophages was upgraded in the lesions (46). It was well illustrated by this experiment that Treg deficiency caused the accumulation of macrophages, which enhanced plaque inflammation, thus exacerbating the process of atherosclerosis (**Figure 3**). Interestingly, great remission of atherosclerotic lesions is achieved by giving Tregs, which was evidenced by the observations of Meng et al. In their study, the results demonstrated that the relative contents of macrophages and lipids in atherosclerotic plaques were reduced, while the contents of smooth muscle cells and collagen were increased, and the index of plaque vulnerability was decreased by nearly 50% after treatment of Tregs in a vulnerable carotid plaque of ApoE^-/-^ mice (52).

### 4.2 Tregs decrease scavenger receptor SRA and CD36 expression to reduce oxidized LDL uptake of macrophages

Macrophage-derived foam-cell formation is a key step for atherogenesis. In the setting of hypercholesterolemia, monocyte-derived macrophages infiltrate the arterial intima to clear retained ApoB-containing lipoproteins (e.g., ox-LDL) and are transformed into lipid-laden macrophage foam cells (60), which persist in the artery wall to promote the formation of plaques (61). If ox-LDL uptake is reduced by macrophages, the process of atherosclerosis will be effectively controlled.

A study by Lin et al. observed the effect of Tregs on ox-LDL uptake in macrophages. Macrophages co-cultured with or without T cells (CD4^+^CD25^+^ T cells or CD4^+^CD25^-^ T cells) were treated with ox-LDL. The results showed that compared with untreated cells or CD4^+^CD25^-^ T-treated cells, foam-cell count and cellular lipid accumulation all decreased markedly in CD4^+^CD25^+^ Tregs. And the expression of CD36 and scavenger receptor A (SRA) that were centrally implicated in the lipid uptake process were downregulated in Treg-treated macrophage foam cells (62). These suggest that Tregs can significantly decrease cholesterol accumulation in macrophages by reducing ox-LDL uptake in these cells, which is due to decreased scavenger receptor SRA and CD36 expression (**Figure 3**).

### 4.3 Tregs transform the phenotype of macrophages to suppress macrophage-derived foam-cell formation

Macrophage polarization is of great significance in the regulation of atherosclerosis. The effects of different types of macrophages on atherosclerosis are described in part 2 of this article. Interestingly, macrophages can alter their phenotype depending on microenvironmental changes during the development of atherosclerosis. Notably, Tregs are involved in changes in the microenvironment and can shift macrophages to a phenotype that is good for suppressing macrophage-derived foam-cell formation (**Figure 3**).

A study by Lin et al. observed the role of CD4^+^CD25^+^ Tregs in macrophage foam-cell formation (62). After co-culture with Tregs, macrophages displayed a decrease in their capacity to produce pro-inflammatory cytokines/chemokines such as tumor necrosis factor (TNF)-α, monocyte chemotactic protein (MCP)-1 and matrix metalloproteinase (MMP)-9. And the production of anti-inflammatory cytokines such as TGF-β and IL-10 was increased (62). Nevertheless, CD4^+^CD25^-^ T-treated cultures showed opposite results, exhibiting an increase in pro-inflammatory cytokines/chemokines and a decrease in anti-inflammatory cytokines/chemokines (62). These results indicated that Tregs could induce the differentiation of macrophages toward an anti-inflammatory phenotype, thereby inhibiting ox-LDL-induced macrophage foam-cell formation. Another study further suggested that CD4^+^CD25^+^FOXP3^+^ Tregs may exert their suppressive functions on pro-inflammatory properties of ox-LDL induced-macrophages via Toll-like receptor 2 (TLR2)-NF-kB signaling pathway (63). This may provide new ideas for an in-depth study of the mechanism of switching macrophage phenotype during atherosclerosis. In 2023, Yu et al. investigated the function of latency-associated peptide (LAP)^+^CD4^+^ T cells, a new class of Tregs in atherosclerosis. Coculturing with CD4^+^LAP^+^ Tregs, monocytes/macrophages display typical features of M2 macrophages (64). Depletion of CD4^+^LAP^+^ Tregs was associated with decreased M2 macrophages and increased Th1 and Th17 cells, characterized by increased unstable plaque and decreased expression of inflammation-resolving factors in both arteries and immune organs (64). In contrast, adoptive transfer of CD4^+^LAP^+^ Tregs induced M2 macrophage differentiation within the atherosclerotic lesions, which was associated with increased collagen and α-SMA in plaques and decreased expression of MMP-2 and MMP-9 (64). These results indicated that CD4^+^LAP^+^ Tregs could protect against atherosclerosis by modulating macrophage polarization.

### 4.4 Tregs enhance the ability of macrophage efferocytosis to promote atherosclerosis regression

Studies have shown that defective resolution and defective efferocytosis play key roles in the progression of relatively benign atherosclerotic lesions into clinically important necrotic plaques (25, 65). The ability of macrophage efferocytosis positively correlates with the regression of atherosclerosis. Proto et al. indicated that Tregs can boost the ability of macrophages to carry out efferocytosis during resolution responses *in vitro* and *in vivo* (66). They found that Tregs derived IL-13 and used them as transcellular mediators to stimulate macrophages to produce IL-10. Then, apoptotic cell engulfment was enhanced by an autocrine-paracrine manner via a Vav1-Rac1-mediated mechanism (66). This was also confirmed by their findings from another angle, which revealed that Treg depletion impaired macrophage efferocytosis during inflammation resolution (66). It should be noted that a large number of studies from both humans and animals suggested Tregs, IL-10, and IL-13 can protect against advanced plaque progression (25, 67–69). The results clearly revealed that Tregs can promote the efferocytosis of macrophages, leading to regression of atherosclerosis (**Figure 3**).

Sharma et al. (9) also nicely demonstrated that Tregs license macrophage efferocytosis, through which regression of atherosclerosis can be well achieved. In their study, there were some excellent findings. First, Tregs participated in the enrichment of M2 macrophages in regressing plaques and licensed their pro-resolving functions, including clearance of apoptotic cells, production of specialized pro-resolving lipid mediators (SPMs), and upregulation of the receptors that sense these mediators of resolution (9). Significantly, these pro-resolving and tissue reparative functions of macrophages failed to activate in the absence of Tregs despite optimal lipid lowering for plaque regression (9). And they observed that the increased expression of receptors for pro-resolving lipid mediators in macrophages was dependent on Tregs during the regression of atherosclerosis (9). These receptors initiated signaling to enhance macrophage phagocytosis of apoptotic cells, reduce pro-inflammatory cytokine, and increase anti-inflammatory cytokine (70). Second, Treg-derived cytokines such as IL-10 and TGF-β can dampen macrophage inflammatory responses, promote alternative activation, and increase efferocytosis (9, 66, 71). Simultaneously, M2 macrophages can also secrete IL-10 and TGF-β, which may, in turn, sustain Tregs (72). Third, they found that Tregs can promote macrophage secretion of resolvin D1 (RvD1) (9), and administration of RvD1 promotes plaque stability by enhanced efferocytosis capacity of macrophages in atherosclerotic plaques in both mice and humans (73).

## 5 Effect of potential factor on Tregs-regulated macrophages in atherosclerosis

Krüppel-like factor 10 (KLF10) is a transcription factor in CD4^+^ T cells, which can regulate the progression of atherosclerosis (74). Wara et al. highlighted the important role of KLF10 in mediating Treg-macrophage coupling in atherosclerosis (74). They chose two types of mice, CD4^+^ T-cell-specific KLF10 knockout mice (TKO mice) and CD4-Cre transgenic mice (also known as wild-type mice (WT mice)), for the experiment. In their study, they found that compared to WT mice, TKO mice exhibited an increase in plaque size as well as higher CD4^+^ T cells and macrophage content (74). Moreover, the plaques in TKO mice showed growth of necrotic cores along with defective macrophage efferocytosis (74). In contrast, adoptive cellular therapy using WT Tregs abrogated the accelerated lesion progression and deleterious effects in TKO mice (74). In addition, RNA-seq analyses indicated that compared to WT lesions, TKO lesions revealed increased chemotaxis and cell proliferation, and reduced phagocytosis (74). These results demonstrated that TKO-Tregs impaired the efferocytosis capacity of macrophages *in vitro* and promoted a pro-inflammatory macrophage phenotype via increased IFN-γ and decreased TGF-β secretion, thus exerting pro-atherogenic activity (74).

## 6 Effects of potential factor on both Tregs and macrophages in atherosclerosis

### 6.1 Mesenchymal stem cells

Bone marrow-derived mesenchymal stem cells (MSCs) play a crucial role in immunomodulation, as they can inhibit the activity of various immune cells, thereby suppressing the immune response *in vitro* and *in vivo* (75–77). Importantly, MSC transplantation attenuated the pathology of atherosclerosis (78). Wang et al. measured that the size of atherosclerotic plaque declined after infusion of MSCs into ApoE^-/-^ mice (78). In addition, the number of CD4^+^CD25^+^FOXP3^+^ Tregs in cultured splenocytes was increased and both mRNA and protein levels of FOXP3 were upregulated in the MSC group (78). To explain, some studies illustrated that FOXP3 controlled the immunosuppressive function of Tregs by regulating the expression of genes such as CTLA-4 and CD25 (79, 80). Previous studies showed that the knockdown of FOXP3 promoted the progression of atherosclerosis in mice, implying its possible atheroprotective function (51). Meanwhile, they found that the formation of macrophage foam cells was inhibited by treatment with MSCs *in vitro* experiments (51). Taken together, MSCs play an atheroprotective role by enhancing the number and function of Tregs and inhibiting the formation of macrophage foam cells.

### 6.2 Interleukin-12p35

Interleukin-12p35 (IL-12p35) is a subunit that constitutes IL-35, which has been considered a functional cytokine of Tregs (81, 82). Huang et al. showed that IL-12p35 deficiency reduced the atherosclerotic plaque in the aortic trees and root, decreased the infiltration of CD4^+^ T cells and macrophages, and increased vascular smooth muscle cells and collagen in the plaques of the ApoE^-/-^ mice (82). These results suggested that IL-12p35 deficiency attenuated atherosclerosis and elicited a stable plaque phenotype, thereby playing a protective role in atherosclerosis (82). Mechanistically, the effect of IL-12p35 deficiency on atherosclerosis depended on the dominant position between the alleviated Th1/Th2 imbalance and the aggravated Th17/Treg imbalance (82).

### 6.3 HCW9302

It was reported that IL-2 contributed to the development and expansion of Tregs (83, 84). HCW9302 is a novel IL-2-based fusion molecule, which can treat atherosclerosis, a new study suggests (85). Zhu et al. observed that HCW9302 caused a marked reduction in atherosclerotic lesion formation in the aortic sinus compared with control mice (85). RNA-seq analysis of the whole aorta demonstrated that the expression of Treg, M2 macrophage, and myeloid derived suppressor cell (MDSC) associated genes were upregulated after HCW9302 treatment (85). Additionally, the expression of inflammation genes (e.g., Pai1, Ccl2, Ccr, CD7Tnfa, Inos1, Trem2, and Prf1) were reduced and the anti-inflammation genes (e.g., Serpinb1c, Mafa, Trim29, Trim72/MG53, Ybx3, and Ptgr1) were elevated (85). These results demonstrated that Tregs, M2 macrophages, and MDSCs reduced inflammation to alleviate atherosclerosis.

### 6.4 MicroRNA-33

It was previously recognized that microRNA-33 (miR-33) and its host genes cooperated to regulate cholesterol homeostasis (86, 87) and reduce atherosclerotic plaque size (88, 89). A study by Rotllan et al. challenged this concept. In the study, they found miR-33 inhibition had anti-atherosclerotic properties that was no effect on the changes in plasma levels of high-density lipoprotein cholesterol (HDL-C) in hyperlipidemic LDL receptor-knockout mice (Ldlr^–/–^ mice) fed a Western diet (90). Coincidentally, consistent results were obtained from the study of Ouimet et al. They revealed that miR-33 regulated macrophage inflammation and demonstrated that miR-33 antagonism was atheroprotective by promoting M2 macrophage polarization and Treg induction to reduce the inflammation of plaque (61). In the process of polarization, macrophage-specific miR-33 deletion induced M2 macrophage polarization-associated gene profile. Anti-miR-33 treatment promoted the accumulation of FOXP3^+^ cells within the adventitia and the plaque intima (61). Of note, anti-miR-33 colocalized with M2 macrophage markers in plaques and was associated with increased plaque macrophage expression of aldehyde dehydrogenase family 1, subfamily A2 (Aldh1a2), which also promoted the expansion of FOXP3^+^ Tregs (61).

### 6.5 Signal transducer and activator of transcription 4

The signal transducer and activator of transcription 4 (STAT4) is a critical regulator of inflammation, playing a pro-inflammatory role in immune-mediated diseases (91). Taghavie-Moghadam et al. investigated the impact of STAT4 on atherogenesis under the circumstances of insulin resistance (IR). It turned out that STAT4 participated in atherogenesis via the support of pro-inflammatory activities of macrophages, regulation of the CD8^+^ Treg/T follicular helper cell axis, and modulation of the local immune response in the aortic wall under conditions of IR and atherosclerosis (92).

### 6.6 Hyperhomocysteinaemia

Hyperhomocysteinaemia (HHcy) is a potent pro-inflammatory factor, accelerating the development of atherosclerosis *in vitro* and *in vivo* (93). Feng et al., for the first time, demonstrated that HHcy-induced Treg reduction in proportion and function may be responsible for HHcy-accelerated atherosclerosis in ApoE^-/-^ mice (94). In the study, the results showed that HHcy attenuated the proportion and suppressive effects of Tregs, with an increase in atherosclerotic lesion area and accumulation of macrophages and T cells in plaques of ApoE^-/-^ mice (94). When the adoptive transfer of Tregs from age-matched normal mice to HHcy ApoE^-/-^ mice, the atherosclerotic lesions were abrogated and the HHcy-induced infiltration of macrophages and T cells was attenuated (94).

## 7 Targeting Tregs and macrophages by potential drugs for atherosclerosis therapy

### 7.1 Formula of Chinese medicine

Si-Miao-Yong-An decoction (SMYAD), a famous formula, is widely used in the treatment of atherosclerosis (95, 96). In 2021, Chen et al. further explored the effects of SMYAD on the pathological changes of atherosclerosis and the differentiation of monocytes, macrophages, and Tregs in ApoE^-/-^ mice (97). The results showed that compared with the model group, the level of TC and LDL-C, the pathological changes of the aortic sinus, and lipid infiltration of the aorta and aortic sinus were all decreased in the SMYAD group, accompanied by downregulation of cluster of differentiation 36 (CD36), SRA1, and lectin-like oxidized low-density lipoprotein receptor-1 (LOX-1) (97). Moreover, the proportions of Ly6Chigh pro-inflammatory monocyte subsets, macrophages, and their M1 phenotypes were reduced in the spleen, while the proportion of Tregs was increased (97). Furthermore, the expression of F4/80 was decreased, while the expression of FOXP3 was increased in the aorta sinus (97). In addition, the levels of serum pro-inflammatory factors IL-1β and IL-18 were decreased (97). These findings suggested that SMYAD can improve the pathological changes of atherosclerosis and inhibit lipid deposition in ApoE^-/-^ mice, which was associated with the inhibition of the differentiation and recruitment of monocytes and macrophages, the promotion of the differentiation and recruitment of Tregs, and the reduction of the secretion of pro-inflammatory factors (97).

### 7.2 C5a receptor-related peptides

Monocyte-to-macrophage differentiation and LDL oxidation play a pivotal role in early atherosclerosis. Lu et al. confirmed that it was effective for the immunization of mice with 2 peptides located at the N-terminus of the C5a receptor (C5aR), either alone (C5aR-P1 or C5aR-P2) or in combination (C5aR-P1 and C5aR-P2) to reduce early atherosclerotic lesions (98). Specifically, C5aR N-terminal peptide immunization can modulate ox-LDL-specific immunity and reduce atherosclerotic lesion formation by inducing specific Treg response and blocking monocyte differentiation into macrophages (98).

## 8 Conclusion

This review discusses the findings of Tregs and macrophages in atherosclerosis in recent years, from mechanisms to clinical significance. First, the crucial role of Tregs and macrophages in atherosclerosis is discussed. Then, the regulatory mechanisms of regulatory T cells on macrophages in atherosclerosis are elaborated, which are mainly manifested in four aspects: (1) Tregs deficiency induces the accumulation of macrophages; (2) Tregs reduce ox-LDL uptake of macrophages; (3) Tregs transform the phenotype of macrophages; (4) Tregs enhance the ability of macrophage efferocytosis. Next, the effects of KLF10 on Tregs-regulated macrophages in atherosclerosis and the effects of MSCs, IL-12p35, HCW9302, MicroRNA-33, STAT4, and HHcy on Tregs and macrophages in atherosclerosis are explained. Finally, targeting the Tregs and macrophages by SMYAD or C5aR-related peptides may offer benefits for atherosclerosis therapy. Studies have indicated that Tregs and macrophages may provide promising targets for the diagnosis, treatment, and prognosis in atherosclerosis.

However, there are some problems with the relevant studies: first, the role of Tregs-regulated macrophages on atherosclerosis has been less studied, which still needs to be strengthened. Second, it has been rarely reported whether it is possible to treat atherosclerosis by influencing macrophages and thus modulating Tregs, in which regard research should be emphasized. Third, numerous studies have shown that simultaneous regulation of Tregs and macrophages can affect atherosclerosis, but the crosstalk between them in this process has not been elucidated in depth and needs to be further explored.
